# Response of marine copepods to a changing tropical environment: winners, losers and implications

**DOI:** 10.7717/peerj.2052

**Published:** 2016-05-24

**Authors:** Li Lee Chew, Ving Ching Chong

**Affiliations:** 1 Institute of Biological Sciences, Faculty of Science, University of Malaya, Kuala Lumpur, Malaysia; 2 Faculty of Applied Sciences, UCSI University, Kuala Lumpur, Malaysia

**Keywords:** Coastal developments, Straits of Malacca, Man-induced effects, Zooplankton, Biodiversity, Species vulnerability, Food webs

## Abstract

**Background.** Climate change concurrent with anthropogenic disturbances can initiate serial changes that reverberate up the food chain with repercussions for fisheries. To date, there is no information available concerning the combined effects of global warming and human impacts on tropical marine food webs. While temperate copepods respond differently to warming and environmental stressors, the extent to which tropical copepods can adapt to rising temperature of already warm waters remains unknown. We hypothesize that sea warming and other anthropogenic disturbances over the long term will have the greatest impact on the copepod community in nearshore waters where their effects are accentuated, and therefore vulnerable and resilient species could be identified.

**Methods.** Zooplankton samples were collected during two time periods (1985–86 and 2014–15) interposed by marked anthropogenic disturbances, and at the same five stations located progressively from inshore to offshore in Klang Strait, Malaysia, following the asymmetrical before-after-control-impact (BACI) design. Copepods were identified to species, and results were interpreted by univariate (ANOVA) and multivariate (PERMANOVA, PCO) analyses of the computed species abundance and diversity measures.

**Results.** Copepod total abundance was not significantly different among stations but higher after disturbance than before disturbance. However, changes in the abundance of particular species and the community structure between time periods were dramatic. Coastal large-bodied calanoid species (e.g., *Acartia spinicauda*, *Calanopia thompsoni*, *Pseudodiaptomus bowmani* and *Tortanus forcipatus*) were the most vulnerable group to disturbance. This however favored the opportunistic species (e.g., *Oithona simplex*, *O. attenuata*, *Hemicyclops* sp., *Pseudomacrochiron* sp. and *Microsetella norvegica*). Small-bodied copepods (e.g., *Paracalanus* sp., *Parvocalanus crassirostris* and *Euterpina acutifrons*) were unaffected. *Centropages tenuiremis* was likely an introduced species. There was no significant loss in species richness of copepods despite the dramatic changes in community structure.

**Discussion.** Sea warming and other human-induced effects such as eutrophication, acidification and coastal habitat degradation are likely the main factors that have altered copepod community structure. The large-bodied estuarine and coastal calanoid copepods are surmised to be vulnerable to eutrophication and hypoxia, while both resilient and opportunistic species are largely unaffected by, or adaptable to, degraded coastal environments and observed sea surface temperature (SST) rise. It is forecasted that SST rise with unmitigated anthropogenic impacts will further reduce large-bodied copepod species the favoured food for fish larvae with dire consequences for coastal fish production.

## Introduction

The dramatic increase in the atmospheric concentration of greenhouse gases, principally CO_2_, through fossil fuel burning is a key factor leading to large-scale warming of the atmosphere and ocean. Investigations in temperate Atlantic and Pacific oceans and the subtropical region of the Mediterranean Sea reveal a consensus that sea surface temperature (SST) rise over the past decades has significantly impacted the species-specific distribution, abundance, phenology and community structure of zooplankton (see Beaugrand et al., 2002; Mackas et al., 2012; Richardson, 2008). There are also studies that have revealed the effects of long-term warming on zooplankton, e.g., the decrease of body size of marine ectotherms at the individual to community level (e.g., Daufresne, Lengfellner & Sommer, 2009; Forster, Hirst & Atkinson, 2012). Changes in zooplankton communities can reverberate up the food chain with serious repercussion on fisheries as has been reported for Atlantic Cod. The fish’s recruitment had dramatically plunged due to sea warming which favored the warm-water, small bodied copepod assemblages (particularly *Calanus helgolandiscus*) over cold-water, large bodied copepod species (particularly *Calanus finmarchicus*) which are the preferred prey (Beaugrand et al., 2003).

Climate change concurrent with anthropogenic impacts (e.g., eutrophication) on the marine environment can lead to other kinds of serial changes in ecosystem function (e.g., Li et al., 2009; Möllmann et al., 2008; Suikkanen et al., 2013). For example, increased SST and dissolved inorganic nutrients aggravate eutrophication that results in the replacement of the larger, more nutritious, diatoms by the smaller, less nutritious or even toxic, microalgae (Hutchins et al., 2007). This is often followed by increased suspended particulate organic matter in estuarine and coastal waters where the high levels of organic matter especially in warm water induce high productivity of heterotrophic bacteria (Berglund et al., 2007; Sarmento et al., 2010). Consequently, the concentration of dissolved oxygen concentration is reduced inducing hypoxia.

To date, there is no information available regarding the impacts of global warming and other anthropogenic disturbances on tropical pelagic food webs. It is unknown how far tropical copepods which constitute an important component of the food web (Chew et al., 2012) can tolerate or adapt to sea temperature rise and other anthropogenic stressors. The ability of tropical copepods to respond to these stressors may also result in contrasting scenarios of change in their community structure, just as in temperate waters. For example, warming may alter the copepod community structure in such a way that species that rely on large nutritious diatoms are eliminated from the system, whereas species that rely on small phytoplankton and heterotrophic microbes thrive in the system (Richardson, 2008). This is because warm water favors small phytoplankton and heterotrophic bacteria (Berglund et al., 2007; Hutchins et al., 2007; Sarmento et al., 2010). Unlike cold temperate waters, it is however difficult to evaluate the *in-situ* or ecological effects of sea temperature rise on marine organisms in tropical waters because of the small temperature change in already warm water.

Klang Strait (in Straits of Malacca), situated off the state of Selangor (Malaysia), has experienced marked environmental changes since the 1980s. Over the past 30 years, the background SST in Klang Strait, attributable to global warming, was reported to have risen 0.58°C, while at the Kapar power station (KPS) specifically, the surrounding water temperature has risen by 1.63°C (Chew et al., 2015). Additionally, loss of mangrove habitats via land reclamation and changes in microbial communities due to deteriorating water quality, in the Klang Strait has been dramatic. The area of the state of Selangor covered by mangrove fell 47% from 28,243 ha in 1980 to 14,897 ha in 2004 (Chong, Sasekumar & Zgozi, 2012). The loss of mangrove is attributed to coastal developments, including expansion of Klang harbor, the Kapar power plant, agriculture, aquaculture, industries and urban settlements (Sasekumar, Then & Moh, 2012). The Klang-Langat river system drains the highly urbanized Klang hinterland where sluggish tributaries at the lower estuary discharge suspended sediment loads generally 1,000–2,000 mg L^−1^, but which exceeded 5,000 mgL^−1^ during freshets (Nelson, 2012). The prevailing poor water quality in nearshore waters as indicated by low dissolved oxygen concentrations close to hypoxia and high total suspended solids (TSS) have also been documented (Lee & Bong, 2006). The Klang Strait has been classified as a eutrophic environment with episodic phytoplankton blooms and high bacteria abundance (Lee & Bong, 2006; Lee, Bong & Hii, 2009; Lim, Lee & Kudo, 2015). While there are no marked changes in deep water fish production, the catch per unit effort of coastal fisheries due to shrimp and bag net catches has dropped dramatically since the mid-1990s despite little change in the fishing effort (see http://www.dof.gov.my/index.php/pages/view/82). The changes in both the environment and fisheries are viewed as symptomatic of anthropogenic impacts that may have altered the food web structure in Klang Strait. Ecological changes and the impact on fisheries are of concern, given that Klang Strait waters account for 40% of the annual fish landings (120,000 tonnes) and 53% of the deployed fishing vessels in the state (Chong, Sasekumar & Zgozi, 2012).

The objective of the present study is to investigate how anthropogenic disturbances in the changing marine environment may alter the assemblage of tropical copepods. We hypothesize that the nearshore coastal environment of Klang Strait has been subject to the greatest impact and that the changing environment over the last 30 years has induced a variety of responses by copepod species reflected by changes in the community structure. We compared and contrasted the copepod community structures in nearshore and offshore waters, and over the two time periods (1985–6 and 2013–4). In addition, we identified the vulnerable and resilient species of tropical zooplankton that could indicate human impacts on marine water quality.

## Materials and Methods

### Study site

**Figure 1:**
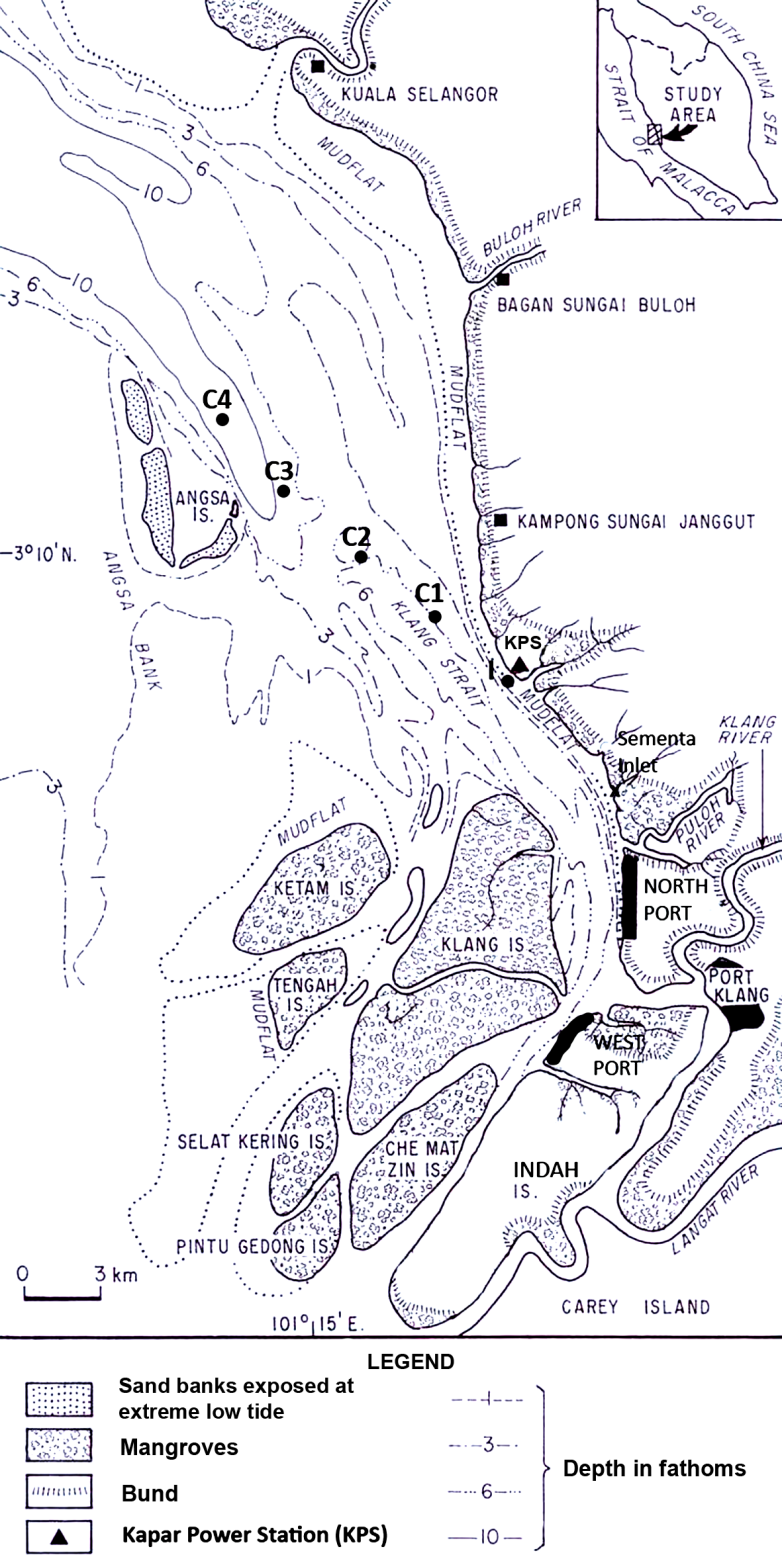
Map of sampling locations in Klang Strait, Peninsular Malaysia. I indicates impacted site closest to Kapar power station (KPS), and C1–C4 indicate control sites along transect line.

Copepods were surveyed in Klang Strait located on the southwest coast of Peninsular Malaysia (Fig. 1). Klang Strait is a narrow, semi-enclosed 70-km long sea channel bounded by extensive sand-mud islands, flats or shoals on its left and right flanks. Currently, the largest tracts of remaining mangrove (ca. 15,000 ha) are located on the Klang islands at the southern part of Klang Strait. The first port facility at Port Klang was established in 1900s and since then three other satellite ports were built around it to cater for the state’s fast expanding trade volumes. The 2,420 MW Kapar power station (KPS) is located at the Kapar estuary situated 24 km north of Port Klang. It has been operating since 1987 to cater to the high power demand in the hinterland, which includes the city of Kuala Lumpur. Six cooling water intake points withdraw a total of approximately 6 million m^3^ seawater daily from Klang Strait and the heated seawater is discharged back into the strait via two outfalls (Azila & Chong, 2010). Temperatures of up to 36°C have been recorded in the thermal effluents but the heat generally dissipates beyond 0.5 km from the outfall (Anton, 1990). Tides in Klang Strait are macrotidal with tidal heights at Mean High Water Spring, Mean Low Water Spring, Mean High Water Neap and Mean Low Water Neap registered at 5.2 m, 1.0 m, 3.9 m and 2.5 m above chart datum (Tide Tables Malaysia, Hydrographic Directorate, Royal Malaysian Navy). Flood and ebb tide streams to the southeast and northwest direction respectively. The maximum tidal velocities of 1.5 m s^−1^ and 0.4 m s^−1^ were recorded during spring and neap tide, respectively (Chong, Sasekumar & Wolanski, 1996).

### Field and laboratory procedures

Abundance and variations in the copepod community at two time periods in Klang Strait were assessed using the asymmetrical before-after control-impact (BACI) sampling design (Underwood, 1992). We examined archived plankton collections from July 1985 to August 1986 (hereafter referred to as before-impact) and recent plankton collections from August 2013 to April 2014 (hereafter as after-impact). These two time periods are separated by almost 30 years, during which the anthropogenic disturbances described above have impacted on the study area.

Sampling stations set 4.5 km apart on a 18-km transect (Fig. 1) were used throughout the study. Organisms nearest to the shore, at station (I) i.e., nearest to Kapar power station or KPS (45 m away), were predicted to be most impacted by the power plant and other anthropogenic disturbances. The other four stations (C1–C4) comprised of controls (Cs) that were located progressively away from I towards offshore. Hence, these control stations were predicted to be progressively less impacted away from shore. The mean water depths at I through C1, C2, C3 to C4 were 15.1 m, 10.8 m, 14.0 m, 14.4 m and 22.5 m, respectively. During before- and after-impact samplings, at least two zooplankton samples were made at each station using the same 45 cm-diameter bongo nets fitted with calibrated flow-meters and twin nets of 363 and 180 µm mesh sizes. Oblique tows from the sea surface to 1 m above the seabed were conducted. Zooplankton samples were preserved with 4% neutralized borax-buffered formaldehyde in seawater for subsequent laboratory enumeration. Environmental parameters including sea surface temperature (SST), salinity, pH and dissolved oxygen were measured *in-situ* with electronic instruments, while after-impact dissolved inorganic nutrients (NH${}_{4}^{+}$, NO${}_{2}^{-}$, NO${}_{3}^{+}$, PO${}_{4}^{3-}$), chlorophyll a (chl. a) and polycyclic aromatic hydrocarbons were chemically measured using an HACH spectrophotometer, Turner 10AU fluorometer or gas chromatograph (Shimadzu GC-17A; Shimadzu, Kyoto, Japan). Except in the discussion, these environment parameters will not be elaborated here since they are reported in Chew et al. (2015).

Only copepods captured by the 180-µm mesh net were enumerated and reported here since small copepods would pass through the 363-µm net. In order to avoid underestimation of large-bodied copepods, each zooplankton sample was gently sieved through stacked Endecott sieves of 1,000 µm, 500 µm, 250 µm and 125 µm mesh, and all size fractions were examined. Copepods from the 1,000-µm fraction were entirely enumerated if the number of individuals was small; otherwise, the zooplankton fraction was split using a Folsom plankton splitter. The enumeration of large copepods (>1,000 µm) was carried out under a stereomicroscope (Leica M125). For smaller size-fractions (500 µm, 250 µm and 125 µm), copepods were subsampled using a 1-ml Stempel pipette and transferred onto a 1-ml Sedgewick rafter cell for enumeration using a compound microscope (Olympus BX50). All adult copepods were identified to species level (Boxshall & Hasley, 2004; Razouls et al., 2012). The abundance of copepods was estimated as number of individuals per cubic meter (ind m^−3^). Since copepodids and nauplii were largely under-sampled by the 180-µm plankton net, they were not considered in the present study.

All field sampling in nearshore waters was conducted with clearance from the Department of Fisheries Malaysia (Permit no. Prk.ML.S.04/32-2 Jld.6(85) and Prk.ML.S.04/32-2 Jld.7(28)) for plankton samplings, and Marine Police Division (Royal Malaysian Police, permit no. 10/7) for conducting work within the harbour limits.

### Data analysis

#### Diversity measures

Species richness (S), Shannon–Wiener diversity index (H’) (Shannon, 1948), Pielou’s evenness (J’) (Pielou, 1969), Simpson dominance index (*λ*) (Simpson, 1949) and average taxonomic distinctness (Δ^+^) (Warwick & Clarke, 1995) were calculated based on 61 identified species. Δ^+^ is a measure of the average taxonomic distance between every pair of species in the sample which precludes the species dominance effect and thus reflects a pure taxonomic relatedness of individuals in the sample (Warwick & Clarke, 1995). Δ^+^ was calculated based on presence–absence data. All diversity measures were computed by using the Plymouth Routines in Multivariate Ecological Research (PRIMER 6) software (Warwick & Clarke, 1995).

#### Univariate analysis

An asymmetrical BACI design ANOVA (Underwood, 1992) was performed using all the diversity measures (i.e., H’, J’, *λ* and Δ^+^), abundance of total adult copepods, and abundance of nine key copepod species (i.e., *Acartia spinicauda, Subeucalanus subcrassus, Paracalanus aculeatus, Paracalanus* sp., *Parvocalanus crassirostris, Bestiolina similis, Oithona attenuata, Oithona simplex* and *Euterpina acutifrons*) to identify any significant spatial and temporal differences. To reveal effects as due to KPS or due to natural spatial and temporal differences, three separate two-way ANOVAs with uneven replicates were performed to compare copepod abundance: (1) among all stations (S) before and after impact, (2) between impacted station (I) and pooled control stations (Cs) before and after impact, and (3) among control stations C1–C4 before and after impact. The significant interactions between before-after (BA) and I vs. Cs in the second ANOVA would reveal any effect of KPS, while the third ANOVA would reveal potential variability due to spatial heterogeneity (Underwood, 1992). A Tukey-HSD test was conducted for significant ANOVA results. Prior to ANOVA, *λ* and abundance data were fourth-rooted so as to fulfill parametric assumptions. The statistical analyses were computed using the Statistica Version 8 software. A further permutation test (shown by funnel plots) was performed on Δ^+^ using PRIMER 6 software to indicate any significant taxonomic deviation from the expected simulated Δ^+^ values against number of species at 95% probability limits (Warwick & Clarke, 2001). All Δ^+^ values were calculated based on a species master list with taxonomically ordered species, i.e., from genus to family to order. The length between each taxonomic level (*ω*) was set at 1 and a given weight of 25.

#### Multivariate analysis

Permutational multivariate analysis of variance (PERMANOVA) analogous to the asymmetrical ANOVA for univariate data was used to detect the significant variations in copepod community structure over space and time. PERMANOVA is a non-parametric test with a pseudo-*F* statistic and *p* value obtained from 999 random permutations performed on the resemblance matrix (Anderson, Gorley & Clarke, 2008). The similarity percentage (SIMPER) procedure was applied to identify the species responsible for the community discrimination. Species exhibiting a large ratio of average to standard deviation [$\bar {\delta }\mathrm{i}$/$\mathrm{SD}(\bar {\delta }\mathrm{i})$] in the dissimilarity measure were considered as good discriminators (Warwick & Clarke, 2001). This criterion was used to select the ‘important’ species for subsequent multi-dimensional scaling. Prior to PERMANOVA and SIMPER procedures, abundance of 61 copepod species was fourth-rooted to normalize the ecological data (Legendre & Legendre, 1998). The Bray–Curtis similarity coefficient (Bray & Curtis, 1957) was used to construct the resemblance matrix among samples. Both PERMANOVA and SIMPER were computed using the PRIMER 6 and PERMANOVA+ software packages.

Principle coordinate analysis (PCO) extracts variance from the resemblance matrix (Bray–Curtis distance) and projects it onto the axes of normally a two-dimensional diagram, and was used to depict the spatial and temporal variations in copepod community structure. As determined by SIMPER, 29 copepod species with $\bar {\delta }\mathrm{i}$/$\mathrm{SD}(\bar {\delta }\mathrm{i})$ ratio of >0.8 and percentage contribution of >2% (see Supplemental Information) were identified as important species and they were selected for PCO. The common (but not abundant) species, *Pontella securifer*, which consistently has $\bar {\delta }\mathrm{i}$/$\mathrm{SD}(\bar {\delta }\mathrm{i})$ ratio of >1 despite a low percentage contribution (<2%) was also included in the PCO. The abundance of the 30 selected species were fourth-rooted before they were subjected to PCO using the PrCoord 1.0 function in the CANOCO 4.5 software (TerBraak & Smilauer, 2002). An additional cluster analysis using average-group linking method of Bray–Curtis similarity was performed to show the average grouping of samples in PCO.

## Results

### Copepod abundance and composition

There was no significant difference in mean total abundance of adult copepods among stations (*p* > 0.05). There was also no significant difference in mean total adult copepod abundance between I vs. Cs (*p* > 0.05). However, mean total abundance of both adult copepods at I and Cs was significantly higher after impact compared to before impact (Table 1 and 2). Nine copepod species dominated the community during both sampling periods accounting for >85% of individuals before and 90% after impact (Table 1). The paracalanids were the most abundant group comprising 52% of the overall adult copepod abundance; four out of the nine key species were representatives from this family i.e., *P. crassirostris* (22%), *Paracalanus* sp. (13%), *P. aculeatus* (10%) and *B. similis* (6%). The oithonids were the most abundant group after the paracalanids with two predominant species *O. simplex* (19%) and *O. attenuata* (7%). Other calanoids (*A. spinicauda* and *Subeucalanus subcrassus*) and harpacticoid (*Euterpina acutifrons*) constituted 2%–6% of total copepod abundance (Table 1).

**Table 1:** Mean abundance (ind m^−3^), percentage abundance (%), frequency of occurrence (FO) and diversity measures of identified copepod species over sampling stations (I, C1, C2, C3, C4) before (B) and after impact (A). <1 denotes less than 1 individual or <1 percent. Symbols in parentheses after species, ‘−’, ‘0’, ‘+’ and ‘R’ denote vulnerable, resilient, opportunistic and rare species, respectively.

Species	I		C1		C2		C3		C4		Overall
	B	A	B	A	B	A	B	A	B	A	
Acartiidae											
*Acartia spinicauda* (−)	495	150	364	39	334	178	185	73	126	65	196
% (FO)	8(100)	1(100)	4(92)	<1(100)	5(100)	2(94)	3(100)	1(100)	3(100)	1(100)	2(99)
*Acartia erythraea* (0)	9	1	7	1	18	78	7	11	27	30	18
% (FO)	<1(31)	<1(13)	<1(23)	<1(19)	<1(60)	1(50)	<1(33)	<1(75)	1(53)	<1(29)	<1(38)
Candaciidae											
*Candacia discaudata* (0)	1	<1	1	2	<1	2	3	44	4	12	7
% (FO)	<1(13)	<1(4)	<1(15)	<1(25)	<1(33)	<1(50)	<1(40)	<1(50)	<1(53)	<1(57)	<1(33)
Centropagidae											
*Centropages dorsispinatus* (0)	39	50	125	159	95	329	53	127	23	106	107
% (FO)	1(63)	<1(46)	2(85)	1(75)	1(67)	3(100)	1(93)	1(100)	1(71)	1(93)	1(77)
*Centropages furcatus* (0)	4	3	1	14	12	26	9	13	13	11	10
% (FO)	<1(25)	<1(8)	<1(15)	<1(50)	<1(60)	<1(63)	<1(53)	<1(81)	<1(71)	<1(64)	<1(48)
*Centropages tenuiremis* (+)		32		52		73		116		48	33
% (FO)		<1(54)		<1(69)		1(69)		1(81)		<1(57)	<1(35)
Calanidae											
*Canthocalanus pauper* (−)	36	4	60	35	72	113	79	329	92	104	89
% (FO)	1(81)	<1(33)	1(85)	<1(75)	1(100)	1(94)	1(93)	4(94)	2(94)	1(100)	1(82)
Eucalanidae											
*Subeucalanus subcrassus* (−)	371	189	336	488	352	731	199	507	257	332	369
% (FO)	6(100)	1(92)	4(100)	4(100)	6(100)	7(100)	4(100)	6(100)	6(100)	3(100)	4(99)
Euchaetiidae											
*Euchaeta concinna* (−)	38	41	18	205	41	136	57	144	46	121	84
% (FO)	1(81)	<1(38)	<1(69)	2(88)	1(93)	1(94)	1(87)	2(94)	1(94)	1(100)	1(81)
Paracalanidae											
*Acrocalanus monachus* (R)						2			3		<1
% (FO)						<1(6)			<1(24)		<1(3)
*Acrocalanus gibber* (−)	90	21	95	81	171	156	109	282	122	192	126
% (FO)	1(100)	<1(46)	1(85)	1(94)	3(100)	2(94)	2(93)	3(94)	3(94)	2(100)	1(88)
*Bestiolina similis* (0)	552	1,053	483	881	293	497	285	179	154	319	500
% (FO)	8(94)	8(100)	6(100)	7(94)	5(87)	5(81)	5(73)	2(69)	4(65)	3(86)	6(85)
*Paracalanus aculeatus* (−)	580	326	802	662	1,112	1,034	9,28	1,477	688	1,432	868
% (FO)	9(100)	2(83)	10(85)	5(100)	17(100)	10(88)	17(100)	17(94)	16(94)	14(100)	10(94)
*Paracalanus* sp. (0)	696	3,368	1,199	1,457	240	473	647	469	394	1,110	1,120
% (FO)	11(94)	24(96)	15(92)	11(100)	4(87)	5(88)	12(87)	5(75)	9(82)	11(86)	13(89)
*Parvocalanus crassirostris* (0)	1,912	2,731	2,445	2,264	2,010	1,900	1,881	1,624	1,421	1,269	1,980
% (FO)	29(100)	20(100)	30(100)	18(100)	32(100)	19(100)	34(93)	18(100)	33(100)	13(100)	22(99)
Pontellidae											
*Calanopia thompsoni* (−)	102	1	50	<1	35	1	18	1	36	2	23
% (FO)	2(94)	<1(21)	1(85)	<1(25)	1(80)	<1(44)	<1(93)	<1(13)	1(82)	<1(7)	<1(52)
*Calanopia elliptica* (R)				<1				<1	1		<1
% (FO)				<1(6)				<1(6)	<1(18)		<1(3)
*Labidocera pavo* (R)					<1						<1
% (FO)					<1(7)						<1(1)
*Labidocera acuta* (R)			<1		<1		<1	<1	<1	<1	<1
% (FO)			<1(8)		<1(7)		<1(7)	<1(6)	<1(6)	<1(7)	<1(4)
*Labidocera bengalensis* (0)	3	<1	<1	5	3	5	5	3	11	4	4
% (FO)	<1(13)	<1(13)	<1(8)	<1(38)	<1(47)	<1(44)	<1(60)	<1(69)	<1(53)	<1(50)	<1(38)
*Labidocera euchaeta* (−)	32	14	93	35	45	50	60	31	46	46	43
% (FO)	<1(94)	<1(33)	1(92)	<1(81)	1(73)	<1(75)	1(87)	<1(81)	1(88)	<1(57)	<1(74)
*Labidocera kroyeri* (R)								<1			<1
% (FO)								<1(6)			<1(1)
*Labidocera pectinata* (0)	4	3	5	3	8	6	6	2	4	8	5
% (FO)	<1(38)	<1(38)	<1(69)	<1(75)	<1(67)	<1(69)	<1(87)	<1(50)	<1(76)	<1(50)	<1(60)
*Labidocera jaafari* (0)	<1	<1	1	<1	<1		<1		<1		<1
% (FO)	<1(31)	<1(8)	<1(31)	<1(25)	<1(7)		<1(20)		<1(6)		<1(12)
*Labidocera* sp. (R)	2	<1				16			<1		2
% (FO)	<1(13)	<1(4)				<1(6)			<1(6)		<1(3)
*Pontella* sp. (R)									<1		<1
% (FO)									<1(6)		<1(1)
*Pontella investigatoris* (R)						<1					<1
% (FO)						<1(6)					<1(1)
*Pontella securifer* (−)	4	<1	1	1	3	<1	2	<1	4		2
% (FO)	<1(63)	<1(13)	<1(69)	<1(25)	<1(87)	<1(13)	<1(60)	<1(13)	<1(82)		<1(41)
Arietellidae											
*Metacalanus aurivilli* (0)	1	12	20	20	15	13	16	5	<1	2	10
% (FO)	<1(6)	<1(21)	<1(23)	<1(19)	<1(47)	<1(13)	<1(27)	<1(19)	<1(6)	<1(7)	<1(19)
Pseudodiaptomidae											
*Pseudodiaptomus bowmani* (−)	79	34	160	80	109	107	102	27	29	21	71
% (FO)	1(88)	<1(63)	2(92)	1(100)	2(93)	1(94)	2(80)	<1(69)	1(82)	<1(36)	1(79)
*Pseudodiaptomus thailandensis* (0)	4	5	49	16			4				7
% (FO)	<1(19)	<1(13)	1(23)	<1(13)			<1(13)				<1(8)
Temoridae											
*Temora discaudata* (R)					<1				<1		<1
% (FO)					<1(7)				<1(6)		<1(1)
*Temora turbinata* (0)	6	27	29	5	28	58	21	72	36	57	33
% (FO)	<1(38)	<1(21)	<1(62)	<1(44)	<1(60)	1(69)	<1(40)	1(69)	1(65)	1(71)	<1(52)
Tortanidae											
*Tortanus barbatus* (0)	1		1	<1	1	3	2	1	1		<1
% (FO)	<1(6)		<1(15)	<1(19)	<1(7)	<1(56)	<1(20)	<1(19)	<1(12)		<1(15)
*Tortanus forcipatus* (−)	143	30	99	44	89	56	43	47	36	25	59
% (FO)	2(88)	<1(67)	1(85)	<1(88)	1(93)	1(94)	1(87)	1(100)	1(76)	<1(93)	1(86)
Lucicutiidae											
*Lucicutia flavicornis* (R)							1				<1
% (FO)							<1(7)				<1(1)
*Lucicutia gaussae* (R)							<1		2		<1
% (FO)							<1(7)		<1(6)		<1(1)
Oithonidae											
*Oithona attenuata* (+)	188	539	217	927	286	1,393	228	1,569	178	1,031	655
% (FO)	3(100)	4(100)	3(100)	7(100)	5(100)	14(100)	4(93)	18(100)	4(100)	10(100)	7(99)
*Oithona brevicornis* (0)	7	11	24	26	19	66	11	58	5	78	29
% (FO)	<1(19)	<1(17)	<1(31)	<1(38)	<1(60)	1(56)	<1(47)	1(56)	<1(29)	1(57)	<1(40)
*Oithona simplex* (+)	212	4,680	280	4,598	75	1,645	190	675	89	2,710	1,688
% (FO)	3(94)	34(100)	3(92)	36(100)	1(73)	16(100)	3(93)	8(94)	2(76)	27(86)	19(91)
*Dioithona rigida* (−)	3		9		3		9		9	2	3
% (FO)	<1(13)		<1(15)		<1(13)		<1(20)		<1(41)	<1(7)	<1(10)
*Oithona plumifera* (0)					1	5	2	1	10	7	3
% (FO)					<1(7)	<1(13)	<1(27)	<1(19)	<1(29)	<1(21)	<1(11)
*Oithona dissimilis* (0)	9	7	11	86		68	4	99	10	6	30
% (FO)	<1(13)	<1(13)	<1(15)	1(19)		1(31)	<1(7)	1(38)	<1(12)	<1(21)	<1(17)
Oncaeidae											
*Oncaea clevei* (0)	4	2		7	10	3	5	7	19	18	7
% (FO)	<1(13)	<1(8)		<1(19)	<1(27)	<1(25)	<1(27)	<1(13)	<1(47)	<1(29)	<1(20)
Corycaeidae											
*Onychocorycaeus pumilus* (R)									<1		<1
% (FO)									<1(6)		<1(1)
*Onychocorycaeus catus* (R)					2	1					<1
% (FO)					<1(7)	<1(6)					<1(1)
*Ditrichocorycaeus affinis* (R)			6		4				1		<1
% (FO)			<1(15)		<1(7)				<1(12)		<1(3)
*Corycaeus speciosus* (R)									1		<1
% (FO)									<1(6)		<1(1)
*Ditrichocorycaeus andrewsi* (−)	117	11	101	25	110	116	65	127	134	104	87
% (FO)	2(94)	<1(42)	1(77)	<1(69)	2(100)	1(94)	1(93)	1(94)	3(100)	1(71)	1(81)
*Ditrichocorycaeus asiaticus* (0)		1			2	2	1	5	1	5	2
% (FO)		<1(8)			<1(7)	<1(31)	<1(13)	<1(6)	<1(12)	<1(7)	<1(9)
*Ditrichocorycaeus dahli* (0)	13		7	3	3	2	3	10	5	6	5
% (FO)	<1(31)		<1(15)	<1(13)	<1(27)	<1(13)	<1(7)	<1(19)	<1(18)	<1(29)	<1(16)
*Ditrichocorycaeus erythraeus* (0)	8			2	3	7	3	4	8	10	4
% (FO)	<1(19)			<1(13)	<1(7)	<1(13)	<1(13)	<1(25)	<1(41)	<1(29)	<1(15)
Macrochironidae											
*Pseudomacrochiron* sp. (+)	12	38	27	53	9	54	35	22	6	45	30
% (FO)	<1(31)	<1(42)	<1(54)	<1(63)	<1(33)	1(81)	1(33)	<1(44)	<1(29)	<1(43)	<1(45)
Clausidiidae											
*Hemicyclops* sp. (+)	8	54	4	31		11	3	11		13	16
% (FO)	<1(19)	<1(63)	<1(15)	<1(25)		<1(25)	<1(7)	<1(19)		<1(14)	<1(21)
Caligidae											
*Caligus* sp. (R)	<1	<1		<1							<1
% (FO)	<1(6)	<1(4)		<1(6)							<1(2)
*Clytemnestra scutellata* (0)	23	24	26	26	21	35	20	28	12	74	28
% (FO)	<1(50)	<1(38)	<1(69)	<1(56)	<1(47)	<1(56)	<1(33)	<1(50)	<1(53)	1(43)	<1(49)
Euterpinidae											
*Euterpina acutifrons* (0)	751	398	1001	576	722	617	303	595	200	452	545
% (FO)	11(100)	3(96)	12(100)	4(100)	11(93)	6(100)	5(93)	7(94)	5(100)	5(100)	6(98)
Ectinosomatidae											
*Microsetella norvegica* (+)	2	15		4	2	6		70		88	18
% (FO)	<1(6)	<1(21)		<1(19)	<1(7)	<1(38)		1(63)		1(57)	<1(21)
Harpacticoid sp. 1 (+)		53	4	3		4	2	5	<1		10
% (FO)		<1(50)	<1(15)	<1(13)		<1(6)	<1(7)	<1(6)	<1(6)		<1(12)
Sapphirinidae											
*Sapphirina gastrica* (R)							<1	<1	<1	1	<1
% (FO)							<1(7)	<1(6)	<1(12)	<1(7)	<1(3)
Monstrillidae sp. (R)		<1									<1
% (FO)		<1(4)									<1(1)
**Total abundance (adults)**	6,559	13,927	8,161	12,916	6,359	10,080	5,607	8,870	4,265	9,963	8,928
**Species richness S**	41	41	39	42	43	44	45	44	50	40	61
**Evenness J’**	0.71	0.57	0.67	0.61	0.67	0.67	0.68	0.68	0.66	0.67	0.71
**Shannon-Wiener H’**	2.18	1.63	2.07	1.90	2.13	2.15	2.13	2.19	2.12	2.08	2.18
**Simpson** *λ*	0.17	0.32	0.21	0.22	0.19	0.18	0.20	0.16	0.21	0.20	0.17
**Taxonomic distinctness** Δ^+^	84.23	86.46	84.39	84.44	83.68	85.53	83.41	85.13	84.34	85.57	84.23

The small copepod *P. crassirostris* was ubiquitous along the strait and its abundance was not significantly affected by space and time (*p* > 0.05 in the ANOVA, Table 2). Although consistently abundant over the past 30 years, there was a stark reduction in percentage contribution after impact, indicating co-occurrence of other dominant species. The percentage composition of *P. crassirostris* before impact ranged from 29% at I to 34% at C3 but dropped to 20% at I and 13% at C4 after impact (Table 1). The other two paracalanids *Paracalanus* sp. and *B. similis*, were not significantly different over time (*p* > 0.05). However, both species were more abundant at I than Cs (*p* < 0.05). Among controls, the abundances of both *Paracalanus* sp. and *B. similis* were significantly higher at C1 than the other three control stations (*p* < 0.01, Table 2).

**Table 2:** Results of asymmetrical ANOVA tests on diversity measures and abundance of total and nine key copepod species.

Variable		Combined tests			Repartitioned tests						
					Between impact and controls (I vs. Cs)			Among controls (Cs)			
		Before-after (BA)	Stations (S)	Interactions (BA × S)	Before-after (BA)	Impact-controls (I vs. Cs)	Interactions (BA × I vs. Cs)	Before-after (BA)	Controls (Cs)		Interactions (BA × Cs)
df		1	4	4	1	1	1	1	3		3
Diversity measures											
J’		B > A*	ns	**	B > A**	ns	**	ns	ns		ns
H’		B > A**	**	**	B > A**	I < Cs**	**	ns	ns		ns
*λ*		ns	ns	**	B < A**	I > Cs*	**	ns	ns		ns
Δ^+^		B < A**	ns	ns	B < A**	I > Cs*	ns	B < A**	ns		ns
Copepod abundance											
Total copepods		B < A**	ns	ns	B < A**	ns	ns	B < A**	ns		ns
*Acartia spinicauda*		B > A**	**	ns	B > A**	I > Cs**	ns	B > A**	ns		ns
*Subeucalanus subcrassus*		ns	**	**	ns	I < Cs**	**	B < A**	ns		ns
*Paracalanus aculeatus*		ns	**	*	ns	I < Cs**	*	ns	ns		ns
*Paracalanus* sp.		ns	**	ns	ns	I > Cs*	ns	ns	**C1^a^**, C2^b^, C3^b^, C4^b^	**	ns
*Bestiolina similis*		ns	**	ns	ns	I > Cs**	ns	ns	**C1^a^**, C2^a,b^, C3^a,b^, C4^b^	**	ns
*Parvocalanus crassirostris*		ns	ns	ns	ns	ns	ns	ns	ns		ns
*Oithona attenuata*		B < A**	*	ns	B < A**	I < Cs**	**	B < A**	ns		ns
*Oithona simplex*		B < A**	**	*	B < A**	I > Cs**	*	B < A**	**C1^a^**, C2^b^, C3^b^, C4^b^	**	ns
*Euterpina acutifrons*		ns	ns	ns	ns	ns	*	ns	ns		ns

**Notes.**

Abbreviations: J’ Pielou’s evenness H’ Shannon-Wiener diversity index (loge-base) *λ* Simpson dominance index B before impact A after impact I impacted station C1–C4 control stations Cs control stations combined df degrees of freedom ns not significant a, b homogeneous group; boldface control station indicate significant highest value, *p < 0.05, **p < 0.01

**Figure 2:**
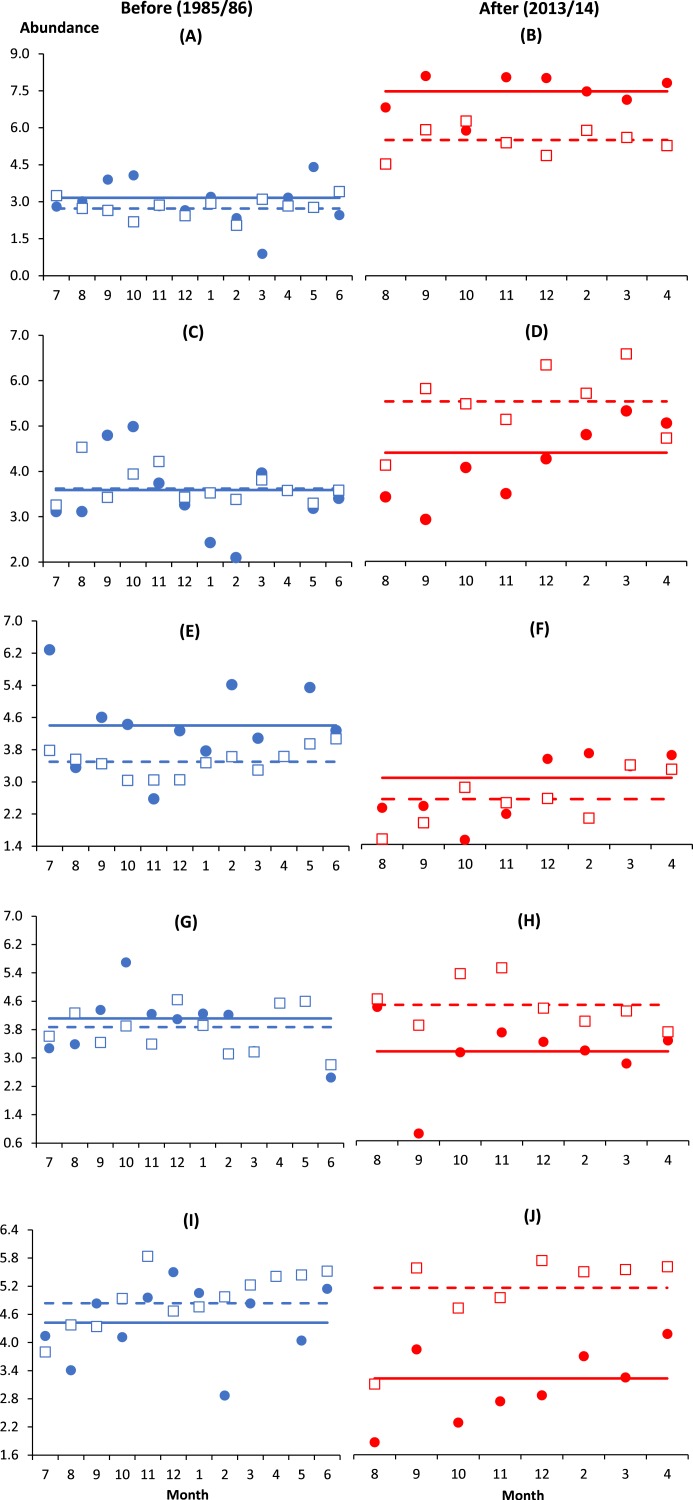
Monthly anomalies of the abundance of five key copepod species (A & B, *Oithona simplex*; C & D, *Oithona attenuata*; E & F, *Acartia spinicauda*; G & H, *Subeucalanus subcrassus*, I & J, *Paracalanus aculeatus*) at impacted (filled circles) and control (empty squares) sites in Klang Strait before (A, C, E, G, I) and after (B, D, F, H, J) establishment of Kapar power station. Anomaly is the vertical distance in number of standard deviations from the mean abundance of all sampling months drawn as solid horizontal line (impacted site, I) or dotted horizontal line (control sites, Cs) on its fourth-rooted value. Horizontal axis indicates the month of sampling (e.g., 7 = July). Vertical axis values of 2, 4, 6 and 8 are equivalent to 16 ind. m^−3^, 256 ind. m^−3^, 1,296 ind. m^−3^ and 4,096 ind. m^−3^, respectively.

Similar to *B. similis* and *Paracalanus* sp., the small-bodied *O. simplex* preferred the coastal water after impact where its abundance was significantly higher at I and C1 as compared to the deeper control stations from C2 to C4 (*p* < 0.01). In contrast, *Oithona attenuata* was least abundant at I and there was no significant difference among control stations (*p* > 0.05). For both *Oithona* species, the interaction effect BA × I vs. Cs (i.e., indicating spatial variability) was only significant after impact (*p* < 0.05) (Table 2 and Figs. 2A–2D). There was a stark increase in abundance of both oithonids after impact (*p* < 0.01, Figs. 2A–2D) since each species constituted less than 5% of total abundance among stations before impact. Except C3, the after-impact mean abundance of *O. simplex* was at least 16 times higher than before impact (Table 1). In particular, more than 34% of copepods at I and C1 comprised of *O. simplex*, which was one of the most abundant copepods in the coastal waters.

Unlike *O. simplex* and *O. attenuata*, the abundance of *Acartia spinicauda* had reduced significantly from before to after impact at both I and Cs (*p* < 0.01, Table 2, Figs. 2E and 2F). The before- and after-impact abundance of *Acartia spinicauda* was significantly higher at I than Cs (*p* < 0.01). There was no significant difference among Cs before and after impact (*p* > 0.05) (Table 2). At I, *A. spinicauda* was the third most abundant copepod species before impact (8%). However, its numerical contribution at I had dropped to merely 1% after impact (Table 1). The other two key calanoid species *S. subcrassus* and *P. aculeatus* were significantly more abundant at Cs than I (*p* < 0.01). Nevertheless, as indicated by the interactions BA × I vs. Cs, their variable numbers at impact and control sites were only significant after impact (*p* < 0.05). Both species were homogenously distributed along the strait before impact but significantly declined in abundance at I after impact (Table 2 and Figs. 2G–2J).

### Copepod diversity measures

**Figure 3:**
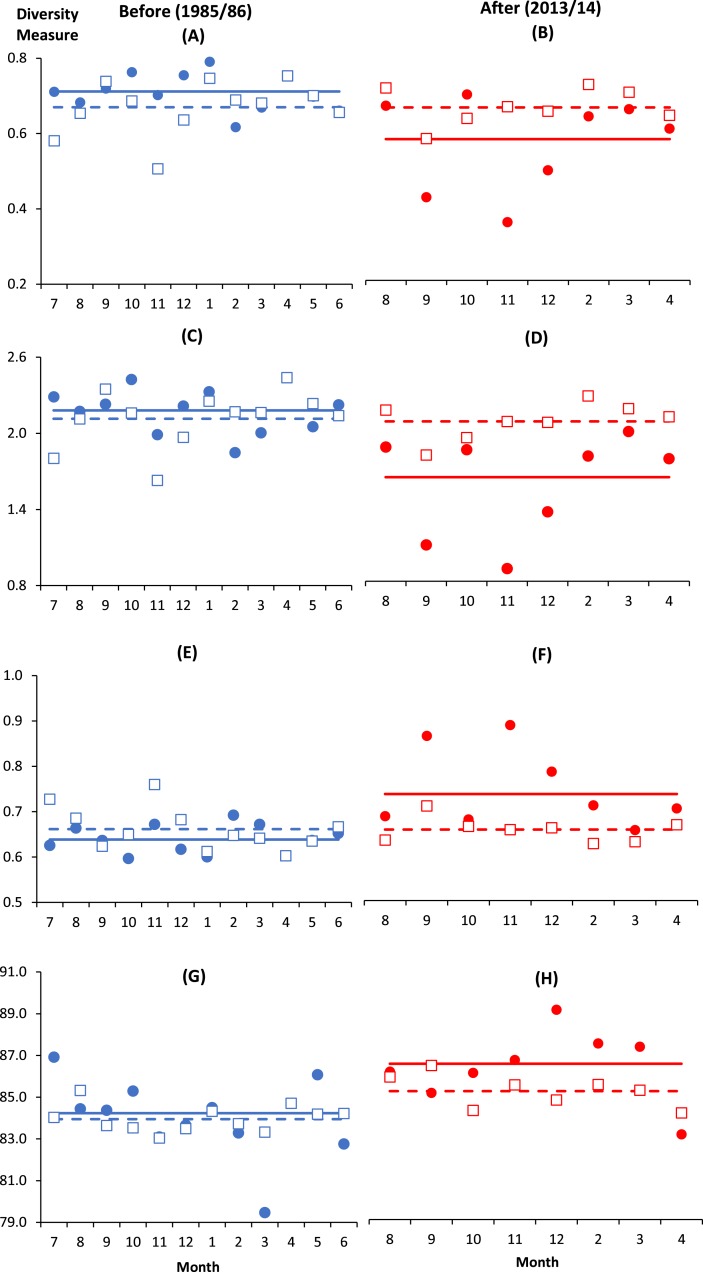
Monthly changes of diversity measures at impacted (filled circles) and control (empty squares) sites in Klang Strait before (A, C, E, G) and after (B, D, F, H) establishment of Kapar power station. (A) & (B)—Evenness index (J’); (C) & (D)—Shannon–Weiner index (H’); (E) & (F)—Simpson dominance index (*λ*); (G) & (H)—Average taxonomic distinctness (Δ^+^). Horizontal lines indicate overall mean for impacted (solid line) and control sites (dotted line).Vertical axis indicates the stated diversity measure. Horizontal axis indicates the month of sampling (e.g., 7 = July).

**Figure 4:**
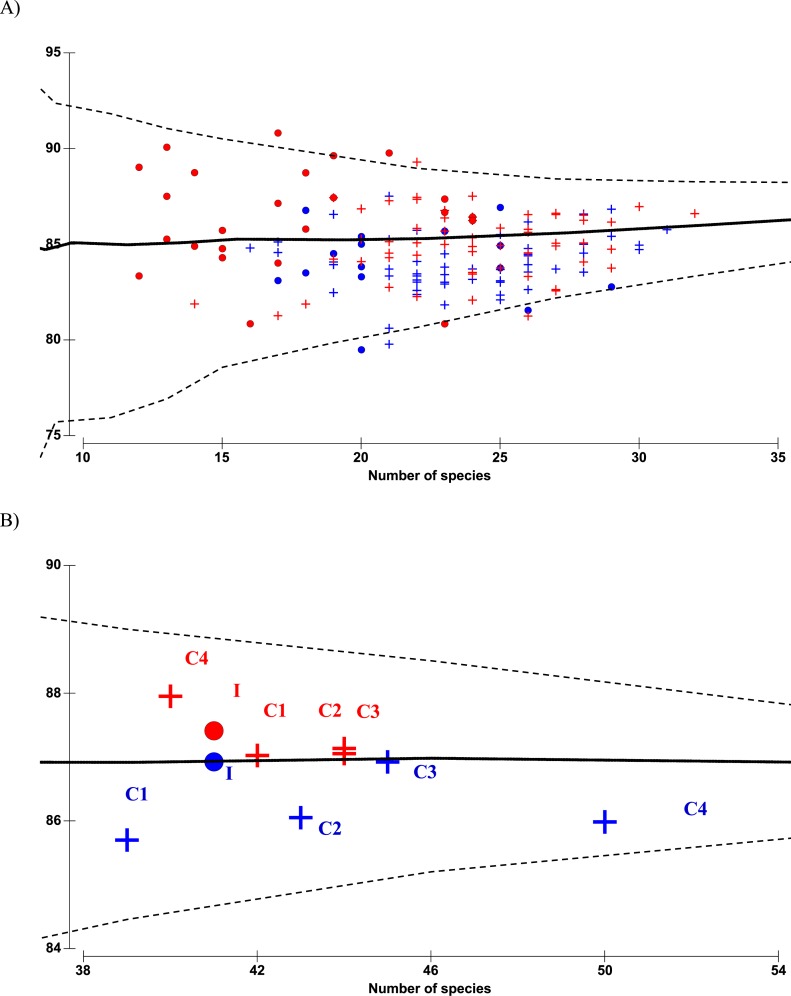
Funnel plots of average taxonomic distinctness Δ^+^ calculated for all samples (A) and pooled samples by site (B) against the number of species. Horizontal solid line indicates overall mean Δ^+^ and dotted lines indicate lower and upper limits of simulated Δ^+^ values. Circle denotes impacted site I, cross denotes controls C1–C4. Before- and after-impact conditions are indicated by blue and red colors, respectively.

There were altogether 57 species recorded before impact compared to 53 species after impact. The difference in species richness at each station before and after impact was small (≤6) except at C4 with much higher number recorded before impact (50 species) compared to after impact (40 species) (Table 1). This is not due to the difference in the number of sampling months between the two time periods since the species rarefaction curves show near maximum number of species achieved after 45 samples or about five months of successive samplings (Data S1). Although a similar species richness before and after impact was recorded at I (41 species), the changes in diversity indices from before to after impact at this station were dramatic. As indicated by the BA × I vs. Cs interaction effects (*p* < 0.05), mean values of the species diversity H’ and evenness J’ at I were significantly higher before than after impact. This was similarly reflected by the species dominance *λ* which was lower before than after impact. The significantly lower J’ and H’ and higher *λ* values at I compared to Cs were only detected after impact. These indices did not show any significant difference between I and Cs before impact (Figs. 3A–3F). The three diversity indices at Cs were consistent over space and time (*p* > 0.05, Table 2). Δ^+^ exhibited larger taxonomic breadth at I as compared to Cs (*p* < 0.05). Δ^+^ was also significantly higher after than before impact at both I and Cs (*p* < 0.01, Table 2). The funnel plots in Fig. 4A showed that most of the after-impact samples (red symbols) fall above the mean Δ^+^(solid line) while the before-impact samples (blue symbols) fall below the mean. However, most of the samples lie within the 95% probability limits (dotted lines) suggesting non-significant deviation of taxonomic distinctness from expected values. The funnel plots based on pooled data indicated that Δ^+^ from all sampling sites were close to expectations even for the after-impact condition (Figs. 4A and 4B).

### Copepod community structure

PERMANOVA tests revealed significant changes in copepod community structure between I and Cs and between before- and after-impact abundance (*p* < 0.01). There were also significant interactions in community measures between BA and I vs. Cs (*p* < 0.01). The before- and after-impact community at I was significantly different from Cs (*p* < 0.01). However, the difference in community structure between I and Cs was more dramatic after impact compared to before impact (Table 3). The three paracalanids *Paracalanus* sp., *P. aculeatus* and *B. similis*, and the oithonid *O. simplex*, were the four species most responsible for community discrimination between I and Cs for both before (cumulative contribution of 17.5%) and after impact (20%) (Data S2). These four species together with the pontellid, *Calanopia thompsoni*, and the corycaeid, *Ditrichocorycaeus andrewsi*, contributed the most to the difference in community structure before and after impact at I.

**Table 3:** PERMANOVA results on copepod community structure in Klang Strait before and after establishment of Kapar power plant.

Variable	Combined tests				Repartitioned tests						
					Between impact and controls				Among controls		
	Before-after (BA)	Stations (S)	Interactions (BA × S)		Before-after (BA)	Impact-controls (I vs. Cs)	Interactions (BA × I vs. Cs)		Before-after (BA)	Controls (Cs)	Interactions (BA × Cs)
df	1	4	4		1	1	1		1	3	3
Pseudo-F	19.57	5.35	2.01		17.72	11.63	4.11		14.32	3.14	1.21
p (perm)	0.001	0.001	0.001		0.001	0.001	0.001		0.001	0.001	0.153
	Average similarity matrix within and between groups										
*BA × I vs. Cs*											
Level	Before				After						
		I	Cs			I	Cs				
	I	72.65			I	62.94					
	Cs	68.97**	67.49		Cs	58.06**	65.74				
Level	I				Cs						
		Before	After			Before	After				
	Before	72.65			Before	67.49					
	After	59.50**	62.94		After	62.85**	65.74				
*BA × Cs*											
Level		C1	C2	C3	C4						
	Before	70.08	69.51	66.98	67.45						
	After	67.56	67.08	65.78	66.64						
	BA	64.74**	64.60**	62.18**	62.83**						
Level	Before						After				
		C1	C2	C3	C4			C1	C2	C3	C4
	C1	70.079					C1	67.56			
	C2	67.96**	69.51				C2	66.14*	67.08		
	C3	66.79**	68.55	66.98			C3	63.48**	66.02	65.78	
	C4	64.66**	68.10	67.18	67.45		C4	64.40**	65.91	66.64	66.64

**Notes.**

Boldface denotes significant at *p < 0.05 and **p < 0.01; df, degrees of freedom.

As indicated by the significant BA x Cs interactions (*p* < 0.05), the copepod community structure among Cs was also significantly different between before and after impact (Table 3). The community at C1 was significantly different from the other three control stations both before and after impact (*p* < 0.05) (Table 3). Again, the four dominant species, *Paracalanus* sp., *B. similis*, *P. aculeatus* and *O. simplex*, contributed the most to the difference in community (Data S2).

**Figure 5:**
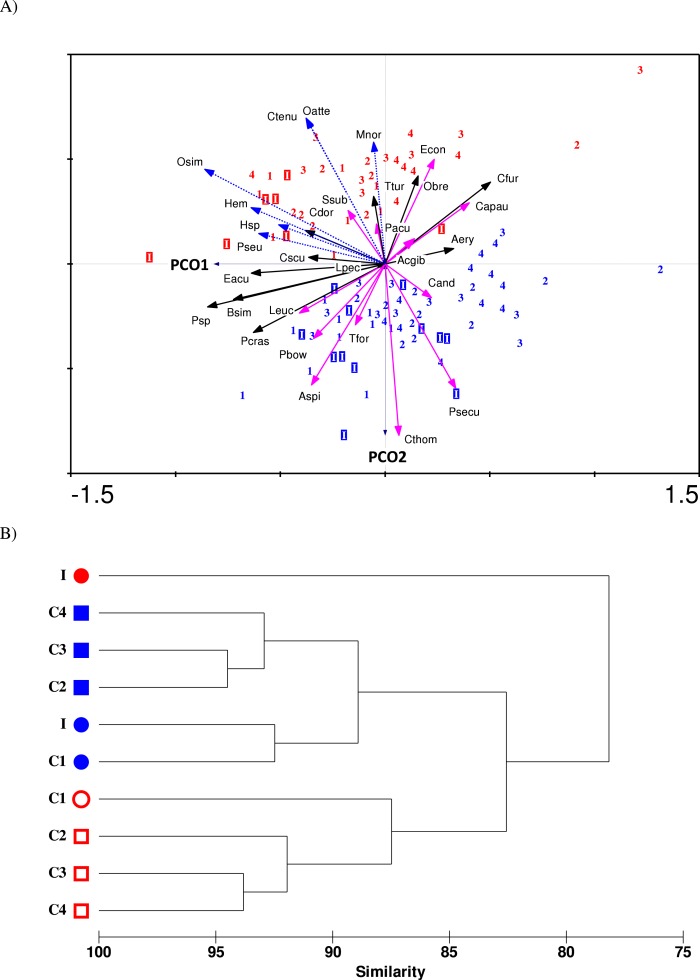
(A) PCO ordination biplot of 30 copepod species and monthly-sampled stations before (July 1985–June 1986, indicated by blue letters) and after power plant impact (August 2013–April 2014, indicated by red letters) in Klang Strait. Stations: letter I denotes impacted station, numbers 1 to 4 denote control stations C1 to C4. Species: vulnerable group I (solid pink arrows)—Aspi *Acartia spinicauda*, Cthom *Calanopia thompsoni*, Tfor *Tortanus forcipatus*, Leuc *Labidocera euchaeta*, Psecu *Pontella securifer*, Pbow *Pseudodiaptomus bowmani*, Cand *Corycaeus andrewsi*; vulnerable group II (solid green arrows)—Acgib *Acrocalanus gibber*, Pacu *Paracalanus aculeatus*, Capau *Canthocalanus pauper*, Ssub *Subeucalanus subcrassus* and Econ *Euchaeta concinna*; resilient group (solid black arrows)—Psp *Paracalanus* sp., Bsim *Bestiolina similis*, Aery *Acartia erythraea*, Cdor *Centropages dorsispinatus*, Cfur *Centropages furcatus*, Ttur *Temora turbinata*, Lpec *Labidocera pectinata*, Obre *Oithona brevicornis*, Pcras *Parvocalanus crassirostris*, Eacu *Euterpina acutifrons*, Cscu *Clytemnestra scutellata*; opportunistic group (dotted blue arrows)—Osim *Oithona simplex*, Oatte *Oithona attenuata*, *Hemicyclops* sp. (Hem), *Pseudomacrochiron* sp. (Pseu), Harpacticoid sp. 1 (Hsp), Ctenu *Centropages tenuiremis*, Mnor *Microsetella norvegica*. (B) Dendrogram from cluster analysis of before-impact (blue symbols) and after-impact (red symbols) zooplankton species (pooled) at impacted station (I) and control stations (C1 to C4). Stations with same symbol (colored circles or squares) were not significantly different in community structure as tested by PERMANOVA.

The ordination biplot from Principal Coordinates Analysis (PCO) shows both spatial (impact and control stations) and temporal (before and after impact) variability in the copepod community structure (Fig. 5A). The first two axes (PCO1 and PCO2) explained a total of 30.1% variance from the abundance data of the 30 selected species. Despite some overlaps, the PCO1 axis defines the inshore-offshore (negative to positive axis) gradient in community structure. Samples from the impacted station (I) are mainly positioned on the negative axis while samples from station C4 (printed 4) are mainly positioned on the positive axis of PCO1. Samples from stations C1 (1), C2 (2) and C3 (3) lie between I and C4. On the other hand, the PCO2 axis clearly distinguishes the before- and after-impact community. Before-impact samples (blue letters) are positioned on the negative axis of PCO2 while after-impact samples (red letters) are oppositely positioned on the positive axis of PCO2. The clustering dendrogram in Fig. 5B summarizes the grouping of samples in Fig. 5A. The after-impact community at I (filled red circle) was distinct from all other communities due to either spatial or temporal effects, indicating a dramatic change in copepod community structure after KPS began operation. Both Figs. 5A and 5B are in agreement with the results from PERMANOVA tests.

The 30 important copepod species subjected to PCO analysis can be categorized into three groups based on a non-parametric Mann–Whitney test that compared the difference between before- and after-impact abundance (*p* < 0.05): (1) “Vulnerable”—those that had significant decline in abundance at I after impact, (2) “Resilient”—those that had no significant difference in abundance at I after impact, and (3) “Opportunistic”—those that had an exceptional or significant increase in abundance after impact. The PCO biplot shows marked spatial and temporal differences among the three groups. In particular, the vulnerable category (pink solid arrows) is separated from the opportunistic group (blue dotted arrows) in opposite directions along PCO2 axis. Four species were highly “vulnerable”: *A. spinicauda* (Aspi), *Calanopia thompsoni* (Cthom), *Tortanus forcipatus* (Tfor) and *Pseudodiaptomus bowmani* (Pbow). All species from the vulnerable group were comparatively large in body size and almost all of them were calanoids (except *C. andrewsi*) and were mostly confined to inshore stations I and C1. Three other species namely *Labidocera euchaeta* (Leuc), *Pontella securifer* (Psecu), and *Ditrichocorycaeus andrewsi* (Cand) were ubiquitous along the strait before impact and had encountered population loss after impact. The five species including *Acrocalanus gibber* (Acgib), *Euchaeta concinna* (Econ), *P. aculeatus* (Pacu), *Canthocalanus pauper* (Capau) and *Subeucalanus subcrassus* (Ssub) were homogenously distributed along the strait before impact and had encountered a significant reduction in abundance at I after impact. However, their abundance remained or even increased at control stations after impact.

The resilient group (black solid arrows) comprised of eight species, which can be further divided into three subgroups: (i) the two abundant paracalanids *Paracalanus* sp. (Psp) and *B. similis* (Bsim), which were not affected by temporal effects but were more abundant in coastal waters (I and C1); (ii) the six calanoid species *Acartia erythraea* (Aery), *Centropages dorsispinatus* (Cdor), *Centropages furcatus* (Cfur), *Temora turbinata* (Ttur) and *Labidocera pectinata* (Lpec) and one oithonid *Oithona brevicornis* (Obre), which did not show any significant change in abundance over time but were more confined to control stations; and (iii) the predominant species *P. crassirostris* (Pcras) as well as the two harpacticoids *E. acutifrons* (Eacu) and *Clytemnestra scutellata* (Cscu), which were homogenously distributed along the strait and not affected by temporal effects.

Seven species were “opportunistic.” Four species *O. simplex* (Osim), *Hemicyclops* sp. (Hem), *Pseudomacrochiron* sp. (Pseu), and Harpacticoid sp. 1 (Hsp), were mostly restricted to coastal waters (I and C1), while three other species, *Centropages tenuiremis* (Ctenu), *O. attenuata* (Oatte) and *Microsetella norvegica* (Mnor) were found mainly in offshore stations (C2–C4). All opportunistic species were low in abundance or absent in 1985/86 but were abundant or common in 2013/14. Interestingly, the calanoid *C. tenuiremis* was not sampled 30 years ago but was common (occurrence of up to 80%) in the 2013/14 survey (Table 1).

## Discussion

In this study we demonstrate a striking alteration in copepod species composition and community structure in the Klang Strait after the impact of the construction and operation of a power station as well as other coastal development along the strait. This dramatic change in copepod community is not likely due to the known periodic cycles of sea surface temperature, e.g., the Indian Ocean Dipole (IOD) and the El Niño-Southern Oscillation (ENSO), since the sampling periods before and after impact were similarly coincident with weak negative IOD conditions (see http://www.jamstec.go.jp/frcgc/research/d1/iod/iod/dipole_mode_index.html) and ENSO-neutral years (see http://ggweather.com/enso/oni.htm). Vulnerable species that were abundant or common before the impact had been replaced by the opportunistic species after the impact. Dominant species belong to the families Paracalanidae (*P. crassirostris, Paracalanus* sp. and *B. similis*) and Oithonidae (*O. simplex* and *O. attenuata*). These species comprised 90% of copepod total abundance at the impacted site in the most recent survey. They are comparatively smaller in body size (<500 µm) than the vulnerable calanoid species *A. spinicauda, C. thompsoni*, *P. bowmani* and *T. barbatus* (>500 µm), which were abundant in the study area before impact (see Table 1).

The increased abundance and occurrence particularly of non-calanoid species explains the increased copepod taxonomic distinctness observed after impact. A similar change in community structure from a community dominated by *Acartia* to one dominated by *Oithona* has been reported from colder regions (e.g., Itoh & Nishida, 2015; Rice, Dam & Steward, 2015; Uye, 1994; Winder & Jassby, 2011). The decline of once-dominant *Acartia* species over decades seems to be a global manifestation, attributed not just to sea warming but also to other anthropogenic impacts. Factors implicated include SST rise due to global warming; e.g., *A. clausii* (Conversi, Peluso & Fonda-Umani, S, 2009) and *A. tonsa* (Kimmel, Boynton & Roman, 2012); eutrophication or/and hypoxia, e.g., *A. tonsa* (Kimmel, Boynton & Roman, 2012) and *A. omori* (Itoh & Nishida, 2015; Uye, 1994); the combined effects of the above factors, e.g., *A. tonsa* (Kimmel, Boynton & Roman, 2012), and shifts in food web structure, e.g., *Acartia* spp. (Winder & Jassby, 2011). However, none of these species are recorded in tropical waters which are populated by their congener, *A. spinicauda* in Klang Strait, both at the control and power plant sites where SST had differed significantly after 30 years of operation of the power plant. Chew et al. (2015) reported that since 1985 the mean SSTs have increased from 29.52 ± 1.10°C to 31.15 ± 0.49°C at the nearest and most impacted station (I) and from 29.30 ± 1.10°C to 29.87 ± 0.64°C at the control stations (Cs). Considering that *A. spinicauda* decreased in abundance not only at the power plant but also at the control site, impact on the species is not solely due to the power plant. Chew et al. (2015) suggested that the eutrophication of Klang Strait waters may also alter the food quality of copepods from large, more nutritious diatoms to small-celled phytoplankton and bacterioplankton. Our results support multiple environmental perturbations rather than a single factor of SST rise (see Chew et al., 2015) as being responsible for the shifts in community structure.

We speculate that eutrophication and hypoxia in the Klang Strait may be significant causal factors in the decline of vulnerable species. Besides *A. spinicauda*, three other vulnerable species (*T. forcipatus*, *C. thompsoni* and *P. bowmani*) which are restricted to estuarine-inshore waters also experienced the most dramatic loss in abundance. Lee & Bong (2006) reported episodic hypoxia due to increased bacterial activity exacerbated by eutrophication and high suspended particulate organic matter in the nearshore waters of Klang Strait. The eggs of these copepod species become vulnerable as they sink to the seabed (Uye, 1994), while adults that migrate up to avoid the hypoxic bottom layer are subject to increasing predation risk (Keister, Houde & Breitburg, 2000; Keister & Tuttle, 2013).

The recent success of oithonids particularly in coastal waters appears to be a global phenomenon. In the present study, the current abundance of *O. simplex* was at least 16 times higher than in 1985/86 at the inshore stations (I , C1 and C2; see Table 1) while abundance of *O. attenuata* had increased at least two fold from past to present. *Oithona* is a good indicator of eutrophication and low oxygen concentration (Richard & Jamet, 2001). The global abundance of *Oithona* species can be related to their physiological and behavioral adaptions which enable them to outcompete other copepods particularly the large-bodied calanoids. These adaptations include their low metabolic and high reproduction rate with increased SST (Almeda et al., 2010; Castellani et al., 2005; Ward & Hirst, 2007), high tolerance to hypoxic waters (Paffenhöfer, 1993; Roman et al., 1993), dietary preference for flagellates which are abundant in eutrophicated waters (Bouley & Kimmerer, 2006; Uye, 1994; Zamora-Terol, Mckinnon & Saiz, 2014), and spawning behavior since they are sac-spawners whose eggs are dissociated from hypoxic sediment (Uye, 1994). Their less conspicuous or small body size (Bouley & Kimmerer, 2006; Svensen et al., 2011) reduces predation pressure. The exceptionally high abundance of *O. simplex* and *O. attenuata* in Klang Strait clearly indicates their much higher reproductive rates over loss rates. The dominance of *O. simplex* in other coastal waters such as at Matang (160 km north of Klang Strait) in the last decade (Chew & Chong, 2011) might be an indication of environmental degradation (Ghaderpour et al., 2014) similar to Klang Strait.

The other small copepod species such as the paracalanids are broadcast spawners which were neither impacted by the power plant nor other anthropogenic activities in Klang Strait. However, it has been reported that the small paracalanids decreased in eutrophicated waters elsewhere due to hypoxic condition (Itoh & Nishida, 2015; Uye, 1994). Consequently, if paracalanids in Klang Strait suffer high mortality due to anthropogenic impacts, this must be compensated for by their continuous reproduction and fast growth rate throughout the year in high temperature and excess food environments (see Escribano et al., 2013; Liang & Uye, 1996). Also, there appears to be reduced predation of small copepods by carnivorous copepods (present study) and fish larvae (C Chu, pers. comm., 2016) whose populations have generally dropped in Klang Strait since three decades ago. Since small paracalanids and oithonids have a relatively similar food spectrum and growth rate (Turner, 2004), one important question begs explanation: why do oithonids rather than paracalanids dominate copepod communities? This could be related to higher egg hatching success or larval survival for sac-spawners compared to broadcast spawners; the removal of oithonid-selective predator(s); or the gradual increase in temperature due to anthropogenic warming has a more positive effect on oithonid compared to paracalanid reproductive or/and growth rates. However, these possibilities need further investigation.

In recent years, two large calanoid species (*Metridia lucens* and *Candacia armata*) that were common in the 1950s have disappeared from Long Island Sound, USA (Rice, Dam & Steward, 2015). The disappearance of other copepod species (*Saphirella indica*, *Pontella andersoni*, *Pseudodiaptomus hickmani*, *Cladostrata brevipoda*, *Laophonte setosa* and *Centropages dorsispinatus*) has been reported in a tropical estuary in India (Bhattacharya et al., 2015). We found no copepod species extirpation from Klang Strait between the mid-1980s and recent years. However, some previously rare species (e.g., *M. norvegica*, *Hemicyclops* sp., *Pseudomacrochiron* sp. and Harpacticoid sp.1) have recently increased in abundance. The harpacticoid *M. norvegica*, which is the most abundant copepod species in the Arctic region where the annual SST does not exceed 6 °C (Arendt et al., 2013), seems to thrive in tropical waters in recent years. The global success of *M. norvegica* is attributable to its ability to utilize suspended organic matter in low food environments (Uye, Aoto & Onbé, 2002) and to adopt both sac- and broadcast spawning strategies (Koski et al., 2014). It has been reported that harpacticoids (*Microsetella* spp.) as well as some poecilostomatoid copepods feed on the discarded larvacean tests (Koski et al., 2007). In the Klang Strait, the abundance of Oikopleuridae has increased at least 10-fold over the past 30 years (Chew et al., 2015). The increased abundance of *Microsetella* and poecilostomatoid copepods may be due to increased food from discarded tests.

We report for the first time the recent occurrence of *Centropages tenuiremis* in Malaysian waters. This species was not observed in the mid-80s. Mulyadi (1998) reported the species as a new record for the Java Sea although he did not observe it in Malaysian waters between 1993 and 1994. Thus, this species is likely introduced into the Klang Strait via ballast water in the late 1990s or later. *Centropages tenuiremis* is one of the dominant species in Xiamen waters, China (Chen et al., 1998; Wu et al., 2007). It is abundant during winter to spring dropping in abundance during summer. Its egg production rate peaked at 14.8°C (Wu et al., 2007). In the Klang Strait, mean water temperatures post-1985 were at or above 29°C, which were much higher than the maximum temperature (25.5°C) recorded by Wu et al. (2007) in Xiamen waters. Therefore, the introduced *C. tenuiremis* in Klang Strait must have physiologically adapted to the local higher temperature environment. It would be interesting to compare the reproductive biology of the two *C. tenuiremis* populations at the extremes of its geographical range.

It is difficult to conclude that a single causal factor had led to the structural alterations of the Klang Strait copepod community. It may be due to sea warming but other factors such as eutrophication, decreased pH and coastal habitat degradation make it more likely a combination of these factors (see Chew et al., 2015). It is recommended that experimental work be conducted in the future to support field results, e.g., on copepod physiological tolerance to SST rise in tropical waters (Lehette et al., 2016), along with long-term monitoring of both zooplankton and environmental parameters. While many studies have reported shifts in copepod community structure over decades of anthropogenic perturbation, Beltrão, Monde & Ueda (2011) did not observe any significant change in community structure in a degraded coastal habitat (Ariake Bay). It has also been reported that power plant discharge did not alter zooplankton community structure in Brazil after 20 years of operation despite reaching a maximum temperature of 36.4°C (Dias & Bonecker, 2008). In the present study, large-bodied copepod species are clearly more vulnerable than small ones to the anthropogenic impacts we investigated. It also seems that the dominant species (*M. norvegica*, *Paracalanus* spp., *Oithona* spp. and *Centropages* spp.) in subtropical and temperate waters are also abundant in tropical waters in recent years. These species are cosmopolitan with high tolerance to environmental perturbations. On the other hand, the geographical range of vulnerable species is comparatively narrower than the cosmopolitan species (Razouls et al., 2012) and they have experienced reductions in different geographical regions due to anthropogenic perturbations. Clearly, the large-bodied estuarine and coastal calanoid copepods (e.g., *A. spinicauda*, *C. thompsoni*, *P. bowmani* and *T. forcipatus*) are losers due to human-induced impacts especially eutrophication and hypoxia. In contrast, both resilient (e.g., *Paracalanus* sp., *P. crassirostris* and * E. acutifrons*) and opportunistic (e.g., *O. simplex*, *O. attenuata* and *M. norvegica*) species are winners in coastal habitats degraded by power plant effluents but also affected by climate change. We predict that in long-term unmitigated coastal developments, economic activities and SST rise will further extirpate large-bodied copepods and replace them with small-bodied species and cosmopolitan species, with further loss in fishery production.

## Supplemental Information

10.7717/peerj.2052/supp-1 Data S1 Chew & Chong (raw data)

10.7717/peerj.2052/supp-2 Data S2 Supplementary material Chew & Chong (supplementary data)

## Additional Information and Declarations

### Field Study Permissions

The following information was supplied relating to field study approvals (i.e., approving body and any reference numbers): The Department of Fisheries Malaysia (Permit no. Prk.ML.S.04/32-2 Jld.6(85) and Prk.ML.S.04/32-2 Jld.7(28)); Marine Police Division, Royal Malaysian Police (permit no. 10/7)

### Data Availability

The following information was supplied regarding data availability: The raw data set has been supplied as Data S1.
