# Supplementary material for: Response of marine copepods to a changing tropical environment: winners, losers and implications
Source: PeerJ. 2016 May 24;4:e2052. doi: 10.7717/peerj.2052 (PMC4888292; doi:10.7717/peerj.2052)
Supplement: Data S2 — Chew & Chong (supplementary data) [file peerj-04-2052-s002.docx]

**Article title:** Response of marine copepods to a changing tropical environment: winners, losers and implications

**Authors:** L. L. Chew & V.C. Chong*

***Corresponding author’s affiliation and email:** Institute of Biological Sciences, Faculty of Science, University of Malaya, 50603 Kuala Lumpur, Malaysia; email: [chong@um.edu.my](mailto:chong@um.edu.my)

**Supplementary data 1**

Rarefraction tests based on the number of cumulative species versus the number of samples, using four methods in PRIMER 6. Abbreviations: Sobs, curve of observed species counts; Chao 1, Chao's estimator based on number of rare species, MM, Michaelis-Menton curve fitted to observed species curve; UGE, calculated species accumulation curve. Dashed-line estimates 52 species or >90% of total species sampled before impact (a) or >98% of species sampled after impact (b) based on 45 samples. The rarefaction curves are drawn by randomly re-sampling the pool of N samples multiple times and then plotting the average number of species found in each sample (1,2, ... N). N = 76 for before-impact condition and 86 for after-impact condition.

**a) Before-impact**

**b) After-impact**

**Supplementary data 2**

The important copepod species contributing to the Bray-Curtis dissimilarity between groups:

a) I vs Cs before impact

| Species | Average abundance (Cs) | Average abundance (I) | $\bar{\delta}$_i_ | $\bar{\delta}$_i_ /SD($\bar{\delta}$_i_) | Contribution percent | Cumulative percent contribution |
| --- | --- | --- | --- | --- | --- | --- |
| *Paracalanus* sp. | 3.73 | 4.64 | 1.58 | 1.29 | 5.08 | 5.08 |
| *Bestiolina similis* | 3.12 | 4.29 | 1.52 | 1.2 | 4.91 | 9.99 |
| *Oithona simplex* | 2.73 | 3.16 | 1.17 | 1.24 | 3.77 | 13.76 |
| *Paracalanus aculeatus* | 4.84 | 4.42 | 1.15 | 1.22 | 3.69 | 17.46 |
| *Euterpina acutifrons* | 4.04 | 4.85 | 1.14 | 1.12 | 3.67 | 21.12 |
| *Centropages dorsispinatus* | 1.98 | 1.61 | 1.11 | 1.31 | 3.57 | 24.69 |
| *Tortanus forcipatus* | 2.28 | 2.88 | 1.07 | 1.31 | 3.43 | 28.13 |
| *Acartia spinicauda* | 3.5 | 4.4 | 1.03 | 1.3 | 3.32 | 31.44 |
| *Calanopia thompsoni* | 1.81 | 2.66 | 1.02 | 1.32 | 3.28 | 34.72 |
| *Pseudodiaptomus bowmani* | 2.42 | 2.42 | 1.02 | 1.27 | 3.27 | 37.99 |
| *Parvocalanus crassirostris* | 6.14 | 6.44 | 1.01 | 1.06 | 3.26 | 41.26 |
| *Euchaeta concinna* | 2.04 | 1.58 | 0.99 | 1.42 | 3.18 | 44.44 |
| *Clytemnestra scutellata* | 1.19 | 1.26 | 0.96 | 1.14 | 3.1 | 47.54 |
| *Labidocera euchaeta* | 2.01 | 2.04 | 0.9 | 1.33 | 2.89 | 50.43 |
| *Temora turbinata* | 1.29 | 0.72 | 0.89 | 1.15 | 2.87 | 53.3 |
| *Canthocalanus pauper* | 2.52 | 1.98 | 0.84 | 1.17 | 2.7 | 56 |
| *Pseudomacrochiron* sp. | 0.91 | 0.78 | 0.82 | 0.94 | 2.65 | 58.66 |
| *Ditrichocorycaeus andrewsi* | 2.79 | 2.95 | 0.8 | 1.09 | 2.58 | 61.24 |
| *Acrocalanus gibber* | 2.91 | 2.77 | 0.78 | 1.29 | 2.52 | 63.75 |
| *Acartia erythraea* | 0.91 | 0.66 | 0.78 | 0.98 | 2.51 | 66.26 |
| *Oithona brevicornis* | 0.93 | 0.44 | 0.74 | 0.91 | 2.38 | 68.64 |
| *Centropages furcatus* | 0.95 | 0.42 | 0.73 | 1.03 | 2.34 | 70.98 |
| *Subeucalanus subcrassus* | 3.87 | 4.12 | 0.71 | 1.37 | 2.3 | 73.28 |
| *Labidocera pectinata* | 1.07 | 0.52 | 0.71 | 1.37 | 2.3 | 75.58 |
| *Pontella securifer* | 0.8 | 0.83 | 0.57 | 1.27 | 1.84 | 77.42 |

b) I vs Cs after impact

| Species | Average abundance (Cs) | Average abundance (I) | $\bar{\delta}$_i_ | $\bar{\delta}$_i_ /SD($\bar{\delta}$_i_) | Contribution percent | Cumulative percent contribution |
| --- | --- | --- | --- | --- | --- | --- |
| *Paracalanus* sp. | 5.3 | 4.14 | 2.22 | 1.15 | 5.29 | 10.86 |
| *Paracalanus aculeatus* | 3.24 | 5.19 | 2.01 | 1.34 | 4.79 | 15.65 |
| *Bestiolina similis* | 4.9 | 3.26 | 1.82 | 1.34 | 4.34 | 19.99 |
| *Euchaeta concinna* | 0.96 | 2.84 | 1.74 | 1.52 | 4.14 | 24.13 |
| *Acrocalanus gibber* | 1.03 | 3.1 | 1.67 | 1.66 | 3.98 | 28.11 |
| *Canthocalanus pauper* | 0.55 | 2.67 | 1.64 | 1.58 | 3.91 | 32.01 |
| *Parvocalanus crassirostris* | 6.31 | 5.68 | 1.6 | 1.33 | 3.82 | 35.83 |
| *Centropages dorsispinatus* | 1.19 | 2.62 | 1.56 | 1.51 | 3.71 | 39.54 |
| *Ditrichocorycaeus andrewsi* | 0.9 | 2.3 | 1.33 | 1.37 | 3.18 | 42.72 |
| *Oithona attenuata* | 4.4 | 5.53 | 1.31 | 1.26 | 3.13 | 45.85 |
| *Subeucalanus subcrassus* | 3.16 | 4.5 | 1.23 | 1.13 | 2.93 | 48.78 |
| *Hemicyclops* sp. | 1.74 | 0.56 | 1.23 | 1.21 | 2.93 | 51.71 |
| *Centropages tenuiremis* | 1.28 | 1.82 | 1.23 | 1.25 | 2.93 | 54.63 |
| *Pseudomacrochiron* sp. | 1.22 | 1.54 | 1.16 | 1.17 | 2.76 | 57.39 |
| *Temora turbinata* | 0.61 | 1.56 | 1.15 | 1.15 | 2.75 | 60.14 |
| *Oithona brevicornis* | 0.44 | 1.56 | 1.15 | 1.01 | 2.74 | 62.88 |
| *Euterpina acutifrons* | 4 | 4.28 | 1.14 | 1.07 | 2.72 | 65.6 |
| *Pseudodiaptomus bowmani* | 1.42 | 1.82 | 1.11 | 1.34 | 2.66 | 68.26 |
| *Labidocera euchaeta* | 0.78 | 1.67 | 1.11 | 1.29 | 2.65 | 70.91 |
| *Tortanus forcipatus* | 1.35 | 2.06 | 1.08 | 1.43 | 2.56 | 73.47 |
| Harpacticoid sp. | 1.5 | 0.17 | 1.07 | 0.96 | 2.56 | 76.03 |
| *Clytemnestra scutellata* | 0.98 | 1.34 | 1.07 | 1.07 | 2.55 | 78.58 |
| *Microsetella norvegica* | 0.51 | 1.08 | 0.91 | 0.87 | 2.17 | 80.74 |
| *Acartia spinicauda* | 3.08 | 2.54 | 0.91 | 1.3 | 2.17 | 82.91 |
| *Centropages furcatus* | 0.19 | 1.14 | 0.86 | 1.04 | 2.06 | 84.97 |

c) Before and after impact at Stn I

| Species | Average abundance (before impact) | Average abundance (after impact) | $\bar{\delta}$_i_ | $\bar{\delta}$_i_ /SD($\bar{\delta}$_i_) | Contribution percent | Cumulative percent contribution |
| --- | --- | --- | --- | --- | --- | --- |
| *Oithona simplex* | 3.16 | 7.52 | 3.39 | 1.8 | 8.37 | 8.37 |
| *Paracalanus* sp. | 4.64 | 5.3 | 1.96 | 1.09 | 4.84 | 13.21 |
| *Calanopia thompsoni* | 2.66 | 0.22 | 1.89 | 2.05 | 4.68 | 17.89 |
| *Ditrichocorycaeus andrewsi* | 2.95 | 0.9 | 1.7 | 1.65 | 4.2 | 22.08 |
| *Paracalanus aculeatus* | 4.42 | 3.24 | 1.54 | 1.15 | 3.81 | 25.89 |
| *Tortanus forcipatus* | 2.88 | 1.35 | 1.54 | 1.53 | 3.79 | 29.68 |
| *Acrocalanus gibber* | 2.77 | 1.03 | 1.5 | 1.64 | 3.71 | 33.39 |
| *Parvocalanus crassirostris* | 6.44 | 6.31 | 1.42 | 1.46 | 3.52 | 36.9 |
| *Bestiolina similis* | 4.29 | 4.9 | 1.39 | 1.24 | 3.42 | 40.32 |
| *Canthocalanus pauper* | 1.98 | 0.55 | 1.29 | 1.49 | 3.18 | 43.5 |
| *Labidocera euchaeta* | 2.04 | 0.78 | 1.28 | 1.59 | 3.17 | 46.67 |
| *Pseudodiaptomus bowmani* | 2.42 | 1.42 | 1.28 | 1.35 | 3.16 | 49.84 |
| *Acartia spinicauda* | 4.4 | 3.08 | 1.28 | 1.23 | 3.16 | 52.99 |
| *Centropages dorsispinatus* | 1.61 | 1.19 | 1.26 | 1.25 | 3.12 | 56.11 |
| *Hemicyclops* sp. | 0.45 | 1.74 | 1.26 | 1.21 | 3.12 | 59.23 |
| *Euchaeta concinna* | 1.58 | 0.96 | 1.24 | 1.24 | 3.07 | 62.3 |
| *Euterpina acutifrons* | 4.85 | 4 | 1.2 | 1 | 2.97 | 65.26 |
| Harpacticoid sp. | 0 | 1.5 | 1.11 | 0.95 | 2.73 | 67.99 |
| *Subeucalanus subcrassus* | 4.12 | 3.16 | 1.11 | 1.1 | 2.73 | 70.72 |
| *Clytemnestra scutellata* | 1.26 | 0.98 | 1.06 | 1.1 | 2.62 | 73.34 |
| *Pseudomacrochiron* sp. | 0.78 | 1.22 | 1.03 | 1 | 2.54 | 75.88 |
| *Oithona attenuata* | 3.59 | 4.4 | 0.99 | 1.6 | 2.45 | 78.34 |
| *Centropages tenuiremis* | 0 | 1.28 | 0.91 | 0.94 | 2.25 | 80.59 |
| *Pontella securifer* | 0.83 | 0.07 | 0.63 | 1.04 | 1.55 | 82.14 |

d) Before and after impact at Controls (pooled C1 to C4)

| Species | Average abundance (before impact) | Average abundance (after impact) | $\bar{\delta}$_i_ | $\bar{\delta}$_i_ /SD($\bar{\delta}$_i_) | Contribution percent | Cumulative percent contribution |
| --- | --- | --- | --- | --- | --- | --- |
| *Oithona simplex* | 2.73 | 5.53 | 2.27 | 1.38 | 6.11 | 6.11 |
| *Paracalanus* sp. | 3.73 | 4.14 | 1.72 | 1.3 | 4.63 | 10.73 |
| *Bestiolina similis* | 3.12 | 3.26 | 1.54 | 1.32 | 4.14 | 14.87 |
| *Oithona attenuata* | 3.62 | 5.53 | 1.47 | 1.56 | 3.96 | 18.82 |
| *Parvocalanus crassirostris* | 6.14 | 5.68 | 1.34 | 1.27 | 3.61 | 22.43 |
| *Paracalanus aculeatus* | 4.84 | 5.19 | 1.32 | 1.07 | 3.56 | 25.99 |
| *Centropages tenuiremis* | 0 | 1.82 | 1.2 | 1.17 | 3.23 | 29.22 |
| *Centropages dorsispinatus* | 1.98 | 2.62 | 1.14 | 1.31 | 3.07 | 32.29 |
| *Calanopia thompsoni* | 1.81 | 0.26 | 1.13 | 1.6 | 3.03 | 35.32 |
| *Pseudodiaptomus bowmani* | 2.42 | 1.82 | 1.11 | 1.38 | 2.98 | 38.31 |
| *Euterpina acutifrons* | 4.04 | 4.28 | 1.07 | 1.1 | 2.88 | 41.19 |
| *Oithona brevicornis* | 0.93 | 1.56 | 1.07 | 1.15 | 2.88 | 44.06 |
| *Labidocera euchaeta* | 2.01 | 1.67 | 1.03 | 1.34 | 2.77 | 46.84 |
| *Euchaeta concinna* | 2.04 | 2.84 | 1.02 | 1.22 | 2.75 | 49.59 |
| *Temora turbinata* | 1.29 | 1.56 | 1.01 | 1.27 | 2.73 | 52.32 |
| *Pseudomacrochiron* sp. | 0.91 | 1.54 | 1.01 | 1.15 | 2.73 | 55.05 |
| *Ditrichocorycaeus andrewsi* | 2.79 | 2.3 | 1.01 | 1.22 | 2.72 | 57.78 |
| *Clytemnestra scutellata* | 1.19 | 1.34 | 0.99 | 1.14 | 2.66 | 60.43 |
| *Acartia spinicauda* | 3.5 | 2.54 | 0.98 | 1.4 | 2.65 | 63.08 |
| *Canthocalanus pauper* | 2.52 | 2.67 | 0.91 | 1.17 | 2.44 | 65.52 |
| *Acrocalanus gibber* | 2.91 | 3.1 | 0.9 | 1.17 | 2.41 | 67.93 |
| *Tortanus forcipatus* | 2.28 | 2.06 | 0.86 | 1.32 | 2.31 | 70.24 |
| *Acartia erythraea* | 0.91 | 0.87 | 0.82 | 1.03 | 2.2 | 72.45 |
| *Centropages furcatus* | 0.95 | 1.14 | 0.8 | 1.21 | 2.15 | 74.6 |
| *Subeucalanus subcrassus* | 3.87 | 4.5 | 0.78 | 1.27 | 2.1 | 76.71 |
| *Microsetella norvegica* | 0.04 | 1.08 | 0.75 | 0.8 | 2.02 | 78.73 |
| *Pontella securifer* | 0.8 | 0.14 | 0.54 | 1.25 | 1.45 | 80.18 |
